# Fine mapping of the major gene *BhHLS1* controlling seed size in wax gourd (*Benincasa hispida*)

**DOI:** 10.3389/fpls.2023.1266796

**Published:** 2023-09-29

**Authors:** Wenrui Yang, Peng Wang, Ting Liu, Lifeng Nong, Zhikui Cheng, Liwen Su, Wenhui Bai, Yan Deng, Zhihao Chen, Zhengguo Liu

**Affiliations:** College of Agriculture, Guangxi University, Nanning, China

**Keywords:** wax gourd, seed size, QTL, *BhHLS1*, N-acetyltransferase HLS1-like protein

## Abstract

**Introduction/Background:**

The seed size of wax gourds is an important agronomic trait; however, the associated genes have not yet been reported.

**Methods:**

In this study, we used a high-density genetic map constructed based on F_8_ recombinant inbred line populations derived from a cross between MY-1 (large seed) and GX-71 (small seed) strains to detect quantitative trait locis (QTLs) for seed-size-related traits in wax gourd over a two-year period.

**Results:**

Two stable QTLs (*qSL10* and *qSW10*) for seed length (SL) and seed width (SW) on chromosome 10 were repeatedly detected over two years (2021–2022). *qSL10* had a phenotypic variation rate of 75.30% and 80.80% in 2021 and 2022, respectively. Whereas, *qSW10* had a phenotypic variation rate of 66.60% and 73.80% in 2021 and 2022, respectively. Further, a single nucleotide polymorphism mutation was found to cause early termination of *Bch10G006400* (*BhHLS1*) translation in GX-71 through sequencing analysis of candidate genes. Based on gene functional annotation and quantitative real-time PCR analyses, *BhHLS1* encoded a probable N-acetyltransferase HLS1-like protein and its expression level was significantly different between parents. Therefore, *BhHLS1* is a major candidate gene associated with a one-factor polymorphism regulating the SL and SW of wax gourds. Finally, based on variation in the *BhHLS1* sequence, a cleaved amplified polymorphic sequence marker was developed for the molecular marker-assisted breeding of wax gourds.

**Discussion:**

Overall, this study is of great significance for the genetic improvement of seed size, verification of gene functions, and cultivation of specific germplasm resources for wax gourds.

## 1 Introduction

The wax gourd [*Benincasa hispida* (Thunb) Cogn. (2n = 2x = 24)] is among the most important Cucurbitaceae family crops. These plants are native to China and East India and are the main vegetable varieties in South China and Southeast Asia (Hu et al., 2022). Seed size is an important agronomic characteristic of plants and is important for plant evolution (Angela et al., 2005). Further, seed size is directly related to the germination rate, germination cycle, seedling viability, survival rate, and fecundity (Soltani et al., 2018; Zhang et al., 2020), and it also exhibits certain correlations with plant seedling and floral organ sizes and stress resistance (Marcela et al., 2020). Wax gourd seeds also exert anti-inflammatory, analgesic, antipyretic, antidiabetic, antioxidant, beauty-associated, and other effects, and have medicinal applications (Qadrie et al., 2009; Gill et al., 2010). Seeds are commercially available products manufactured by seed companies. In general, large seeds produce larger seedlings with better growth and stronger resistance than small seeds, whereas small seeds have advantages in seed propagation, which affects the reproductive ability of the offspring (Angela et al., 2005; Wu et al., 2006). Therefore, the fine mapping and cloning of major genes controlling the seed size of wax gourds have important theoretical and practical significance for improving crop germplasm resources, increasing yields, and improving their economic value. Seed size is determined by the coordinated growth of zygotic tissues, such as the embryo and endosperm, and maternal tissues, such as ovules (Li et al., 2019a). However, this coordinated growth is a complex process. Additionally, the involvement of some signaling pathways in regulating seed size has been reported in model plants such as *Arabidopsis* and rice (*Oryza sativa* L.). These include the ubiquitin-proteasome, HAIKU, plant hormone, mitogen-activated protein kinase signaling, G protein signaling, and transcriptional regulatory factors-related pathways (Zhao et al., 2016; Zhou et al., 2017; Li et al., 2019a; Tian et al., 2021; Wu et al., 2022a; Wu et al., 2022c). Moreover, gene mapping of seed size-related traits in major Cucurbitaceae crops (pumpkin, watermelon, cucumber, melon, etc.) has also been performed. For example, Wang et al. (2020) used an F_2_ population to construct a high-density genetic map using specific-locus fragment sequencing, and the main quantitative trait loci (QTLs) of seed width (SW) and seed length (SL) were detected in pumpkin. Therefore, it was speculated that the genes encoding the VQ protein, E3 ubiquitin-protein ligase, and proteins containing F-box and LRR domains might be related to SW and SL in pumpkins. Moreover, Li et al. (2018) identified three candidate genes based on fine localization according to the dominant QTL *qSS6-*controlling watermelon thousand-grain weight, grain length, and grain width, including a homologue of the *SRS3* gene in rice that regulates seed size. Further, Wang et al. (2021) performed gene knockout and transcriptome analyses based on the candidate gene *ClBG1* in watermelon, revealing that it might regulate the transcription of cell cycle- and signaling-related genes, mainly by regulating abscisic acid (ABA) content, thus playing a key role in determining the size of watermelon seeds. Through genome-wide association analysis of 197 watermelon samples, Gong et al. (2022) concluded that the *Cla97C05G104360* and *Cla97C05G104380* genes might be involved in ABA metabolism and could be key genes regulating the seed size of watermelon. Based on a simple sequence repeat high-density map constructed using a recombinant inbred line (RIL) population, Wang et al. (2014) detected 14 total QTLs related to cucumber SL, SW, and 100-seed weight (100-SW) in two years, and these were found to be distributed on chromosomes 2, 3, 4, 5, and 6, which could explain the observed phenotypic variation rate of 7.4%–28.3%. In addition, Zhang et al. (2022) used F_2:3_ families and an F_2_ segregation population to repeatedly detect two QTLs related to melon seeds in two environments through regional linkage analysis and RNA-seq. The *EVM0009818* gene, involved in cytokinin-activated signal transduction, was finally identified as a candidate gene for controlling SL and SW. In summary, the genetic research on seed size traits in Cucurbitaceae is not sufficient, and most studies involved the initial mapping. Therefore, identifying genes that regulate seed size is a challenging issue that needs to be solved. To date, there are few reports on the related characteristics of wax gourd seeds. Luo et al. (2022) identified *Bhi04G000544* (*BhYAB4*), encoding the YABBY transcription factor, as a candidate gene regulating the seed shape of the wax gourd based on a genome-wide association analysis and genetic mapping. However, genetic research on seed size traits of wax gourd remains unavailable.

In this study, we constructed a high-density genetic linkage map of wax gourd (unpublished) based on resequencing data of parents and RIL (105) populations. Combining this map with phenotype data, we performed QTL analysis for seed-size-related traits (SL and SW) in wax gourd in 2021 and 2022. The purpose of this study was to identify and clone candidate genes that control seed size in wax gourds and to develop molecular markers that could provide a theoretical basis for the associated genetic mechanisms in wax gourds as well as for future breeding.

## 2 Materials and methods

### 2.1 Population construction, phenotype statistics, and data processing

The wax gourd materials used in this study were a RIL (F8) population derived from a cross between MY-1 (large-seed) and GX-71 (small-seed). The experimental population comprised 107 genealogies, including parents, which were planted at the Yong’an Wax Gourd Experimental Field in Nanning, Guangxi (108°51’ E, 22°48’ N), in July 2021 and 2022. We adopted a field design of randomized blocks with three replicates. There were 10 plants in each plot, with a spacing of 0.5 × 1.5 m. The plants were cultivated in an open field, and cultivation management was carried out according to the method used for general production. All materials in this experiment were provided by Nanning Kenong Seedlings Co., Ltd. (Guangxi, China).

The plants were pollinated regularly and marked, and seeds were sampled from 105 families at 50 days after pollination (DAP); three fruits were randomly selected from each plot for mixed seed washing and drying. Seeds of both parents (at 4, 5, 6, 7, 8, 9, 10, 12, 14, 16, 18, 20, 25, 30, and 50 DAP) were sampled, and their SL and SW were measured using Vernier calipers; 20 full seeds were selected each time. The descriptive statistics, F-test and analysis of variance of phenotypic data were performed using SPSS V27.0 (SPSS Inc, Chicago, Illinois, USA) and Microsoft Excel 2019. GraphPad Prism 9 was used to determine the significant difference and to perform correlation analysis and frequency distribution analysis of the population data.

### 2.2 Histological analysis

To compare the cytological characteristics of the seeds between the two parents, seeds with the same growth based on the maximum cross-section of wax gourd fruit (30 DAP) were sampled. They were immediately fixed in a formalin/acetic acid/alcohol fixative solution with 70% ethanol for 24 h and dewaxed using a dehydrator (Excelsior AS; Thermo Fisher Scientific, Waltham, MA, USA). Wax blocks were sliced into sections using a paraffin slicer (Histocore Arcadia h; Leica Biosystems, Nussloch, Germany), and 4–7 μm thick tissue slices were prepared using a slicer (RM2235; Leica). Subsequently, the sections were rehydrated and stained using the safranin O/fast green staining method, and finally dewaxed with xylene. Tissue sections were scanned using a fluorescence microscope (AxioVert.A1; Zeiss, Oberkochen, Germany), and the images were observed using the NDP.view.2 software (https://nanozoomer.hamamatsu.com/eu/en/products/product-for-research/U12388-01.html). Cell sizes and numbers were determined using the ImageJ software (https://imagej.net/downloads), and the experiment was repeated three times.

### 2.3 DNA extraction and whole genome resequencing

The genomic DNA of the parents and the RIL population was extracted from leaf materials using a Plant Genome DNA Extraction Kit (Solarbio Science, Beijing, China), following the manufacturer’s instructions. After the samples had passed the genomic DNA quality test, the library was constructed. Based on library quality control, 105 families of the RIL population were sequenced at a depth of 20× using the DNBSEQ-T7 platform. The results of 30× deep sequencing of the parents have been reported in a previous study (Ma et al., 2021).

### 2.4 QTL mapping analysis

The resequencing data of the parents and 105 inbred lines were compared with the wax gourd reference genome (GX-19; unpublished), variations were detected, and a high-density genetic linkage map (unpublished) was constructed according to the method proposed previously (Xie et al., 2010). The genetic map consisted of 1,256,985 markers, divided into 956 bins and 12 linkage groups (LGs). The total and average map distances were 1357.145 and 0.002 cM, respectively, and the maximum interval between adjacent markers was 19.647 cM. QTL mapping was performed using high-density genetic maps and phenotypic data. The composite interval mapping (CIM) method was used to locate QTLs using the QTL Cartographer (version1.17j) software (https://brcwebportal.cos.ncsu.edu/qtlcart/), and the QTL threshold was determined based on permutations (1000 times, paired 0.05). After determining the threshold, the QTLs related to SL and SW in this range were determined and named based on well-established guidelines (Mary, 1997).

### 2.5 Extraction of RNA and functional prediction, cloning, and sequencing of candidate genes

The corresponding gene function annotations were searched in the existing reference genome (GX-19). According to the coding sequences (CDSs) of the genes, the primers for cloning (**Supplementary Table S1**) were designed using the Premier 5.0 software. Total RNA was extracted from MY-1 and GX-71 seeds using the Eastep^®^ Super Total RNA Extraction Kit (Promega, Beijing, China), following the manufacturer’s instructions. The first-strand cDNA was synthesized using the 5× All-in-One Master Mix kit, and 2× Phanta^®^ Max Master Mix (Vazyme, Nanjing, China) was used for PCR amplification. The PCR products were detected using 1.2% agarose gel electrophoresis. Thereafter, amplicons were recovered from the bright target bands and purified using an Axyprep DNA gel extraction kit (Axyprep, Union City, CA, USA). A zero-background pTOPO-Blunt cloning kit from Aidlab (Beijing, China) was used to construct an expression vector, following manufacturer’s instructions, which was then transfected into Trans5α competent cells (*Escherichia coli)*. Finally, we selected a single clone for sequencing. The sequencing results were provided by Shanghai Shenggong Biotechnology Co. Ltd. (Shanghai, China), and were compared based on DNA and amino acid sequences using the DNAMANV.8 software.

### 2.6 Quantitative real-time PCR (qRT-PCR) of candidate genes

Quantitative real-time PCR was used to analyze the specific expression of candidate genes. We extracted total RNA from seeds at different stages and plant tissues (stems, leaves, and male and female flowers) at the flowering stage, and the RNA was reverse transcribed into cDNA using the reverse transcriptase RT Master Mix (Takara, Beijing, China), following the manufacturer’s protocol. The primer sequences (**Supplemental Table S1**) of *CAC* (*Bch05G003650*) and candidate genes were designed using Premier 5.0. The premixed SYBR Green quantitative PCR system was used, and *CAC* (*Bch05G003650*) was used as the internal reference gene, with three replicates. An Applied Biosystems7500 real-time quantitative PCR system (Foster City, CA, USA) was used for qPCR analysis, and the relative expression was determined based on the 2^−ΔΔCT^ method (Livak and Schmittgen, 2001), with *CAC* as the internal control; significant differences in relative expression were analyzed using GraphPad Prism 9.

### 2.7 Phylogenetic tree construction

We analyzed the *BchHLS1*-encoded protein sequence using NCBI BLAST (NCBI, Bethesda, MD, USA). Further, its homologous protein sequences in Cucurbitaceae, tomato, rice, and *Arabidopsis thaliana* were downloaded from NCBI in FASTA format (**Supplementary Table S2**). A neighbor-joining phylogenetic tree was constructed using MEGA 11.0, and the confidence level was determined by bootstrap analysis. The number of bootstraps was set to 1000. These files were viewed and edited using MEGA 11.0.

### 2.8 Molecular marker-assisted selection test

Parents, F_1_ plants, and 60 extreme wax gourd germplasm resources, including 28 large seeds and 32 small seeds, were used for marker-assisted selection tests. Based on the CDS differences in candidate genes among parents, a cleaved amplified polymorphic sequence (CAPS) marker was developed for the variation site using the online website dCAPS Finder2.0 (http://helix.wustl.edu/dcaps/dcaps.html). Premier5.0 software was used to design primer sequences, which are listed in **Supplementary Table S1**. DNA was extracted from the tested materials, and following PCR amplification, the products were digested with QuickCutTM TspR1 (NEB, Beijing, China) at 65 °C for 15 min according to the manufacturer’s instructions. The digested products were visualized on 8% non-denaturing polyacrylamide gel electrophoresis and photographed after silver staining. The germplasm resources for the experiments were provided by our research group (**Supplementary Table S3**).

## 3 Results

### 3.1 Phenotypic analysis

**Figure 1:**
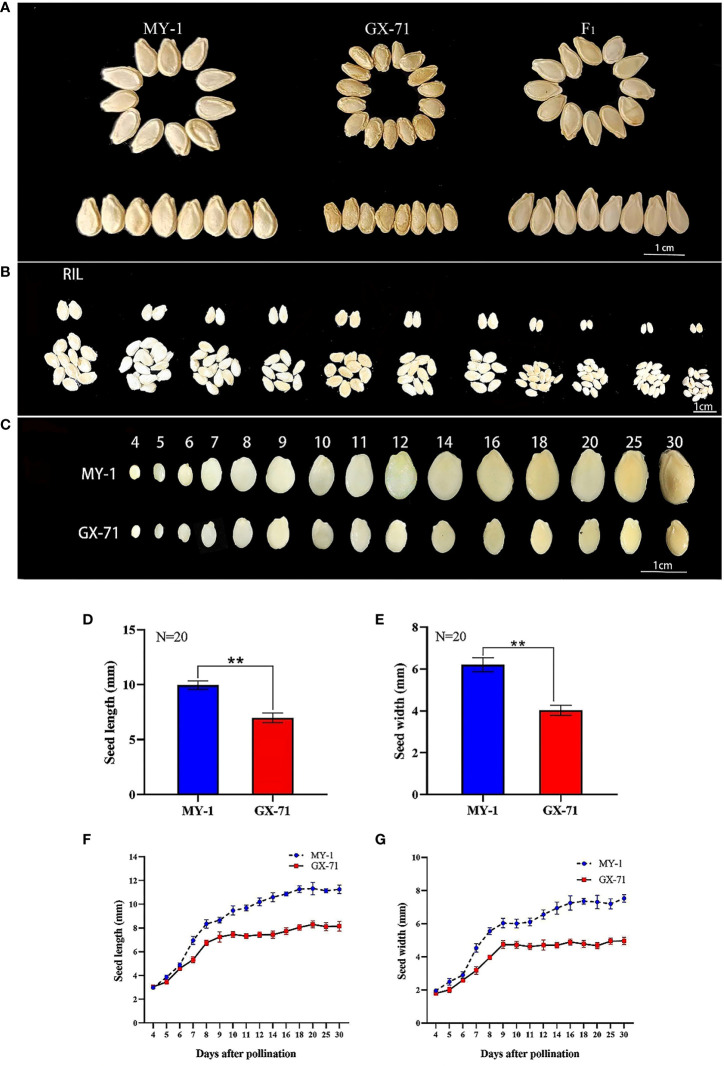
Analysis of phenotypic differences in wax gourd seeds. **(A)**: Phenotypic characteristics of mature seeds of GX-71 and MY-1, scale bar = 1 cm. **(B)**: Phenotypic characteristics of mature seeds in some families of the recombinant inbred line (RIL) population, scale bar = 1 cm. **(C)**: Phenotypic characteristics of seed size of GX-71 and MY-1 at different growth stages, scale bar = 1 cm. **(D, E)**: Significant differences in seed length and width between GX-71 and MY-1. **(F, G)**: Growth curves of seed length and seed width for GX-71 and MY-1 (N: the number of seeds measured; Student’s t-test; **P < 0.01, values are presented as the mean ± SE).

There were significant differences in SL and SW between GX-71 and MY-1 (**Figures 1A, D, E**). Accordingly, GX-71 and MY-1 were considered suitable parents for constructing RIL populations to perform QTL mapping and mine QTLs related to seed size. The SL and SW of the RIL population indicated consistent phenotypic and transgressive segregation across different years (**Figure 1B**; **Supplementary Table S4**). The seed-size-related traits of RIL (105) population showed bimodal continuous distribution and similar trends in two years, so seed size may be mainly regulated by a few major QTLs (**Supplementary Figure 1**). During the seed growth and development processes, the growth patterns of MY-1 and GX-71 in terms of SL and SW were essentially the same, but the seeds of MY-1 were larger than those of GX-71 at all-time points. In the early stage of seed development (4–6 DAP), there was a slight difference between MY-1 and GX-71 seeds; meanwhile, 6–8 DAP and 6–9 DAP were the periods with the highest SL and SW growth rates, respectively. SL and SW tended to be stable at 9 DAP in GX-71, whereas the SL and SW grew slowly at 9–14 DAP and stabilized at 14–30 DAP in MY-1 (**Figures 1C, F, G**). These results showed that the rapid growth period of seeds occurred during the early stages of seed development and that the seed size characteristics were formed in the early stages of development. Nutrient accumulation in seeds was completed in the later stages of growth, and the maturity period of large seeds lagged compared to that of small seeds.

### 3.2 Histological analysis

**Figure 2:**
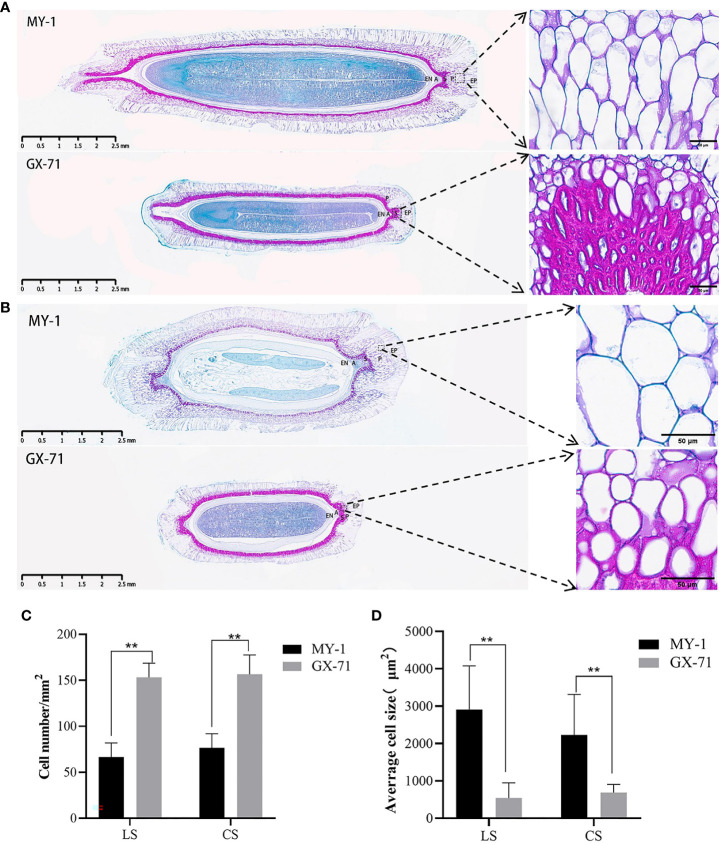
Microscopic, cell size, and cell number analysis of paraffin sections of wax gourd seeds 30 days after pollination (DAP). **(A)**: Longitudinal paraffin sections of seeds of GX-71 and MY-1 at 30 DAP. **(B)**: Paraffin sections of GX-71 and MY-1 seeds at 30 DAP based on cross-sections. **(C)**: Analysis of the differences in the numbers of GX-71 and MY-1 cells (LS: longitudinal section, CS: cross-section). **(D)**: Analysis of cell area differences between GX-71 and MY-1 (LS: longitudinal section, CS: cross-section; panoramic view, scale bar = 2.5 mm; local picture, scale bar = 50 μm; EP: epidermis, P: parenchyma, S: sclerenchyma, A: ventilatory tissue, EN: endodermis; Student’s t-test, **P < 0.01, values are presented as the mean ± SE).

To further explore the mechanism underlying seed size determination, paraffin sections of MY-1 and GX-71 specimens at 30 DAP, based on both transverse and longitudinal planes, were studied. The seed coat was found to be composed of five layers of cells: the epidermis, parenchyma, sclerenchyma, aerenchyma, and endodermis. MY-1 and GX-71 seed coat cells were significantly different in size and arrangement, with MY-1 having large and long parenchyma and narrower, tightly arranged sclerenchyma and GX-71 having small and round parenchyma and thicker sclerenchyma (**Figures 2A, B**). Moreover, the differences in cell size and cell number in the seed coat parenchyma between parents were highly significant (**Figures 2C, D**). Interestingly, the cotyledons of GX-71 were curved apically (**Figure 2A**).

### 3.3 QTL mapping analysis

**Figure 3:**
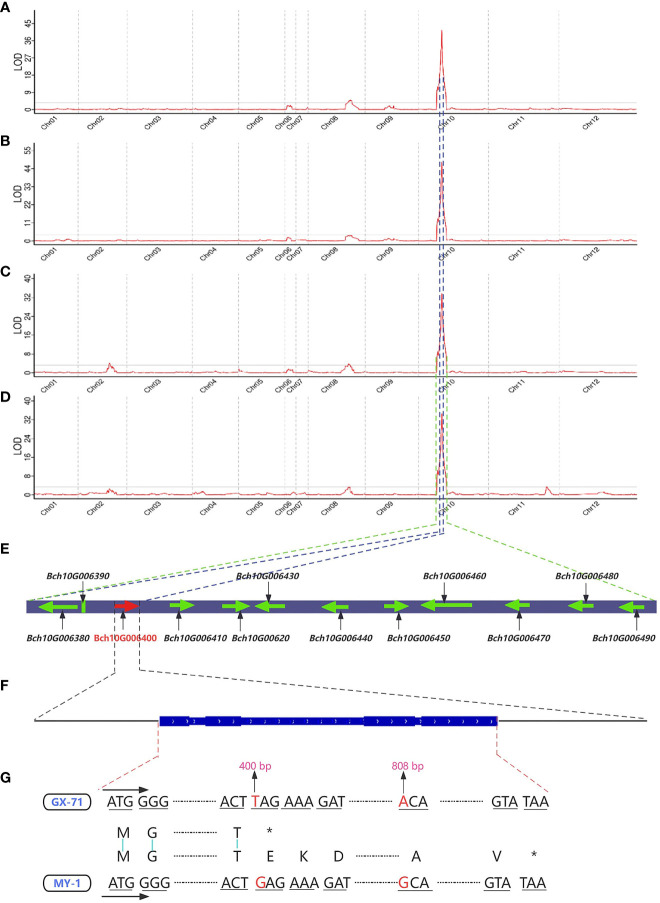
Gene mapping of traits related to seed size in the wax gourd. Distribution of LOD (limit of detection) values for wax gourd seed length in 2021 **(A)** and 2022 **(B)**. Distribution of LOD values for wax gourd seed width in 2021 **(C)** and 2022 **(D)**. **(E)**: Candidate genes located within the quantitative trait loci (QTL) mapping interval (the genes contained within the blue dashed lines are candidate genes within the *qSL10* interval, whereas the genes contained within the green dashed line are candidate genes within the *qSW10* interval; the arrow represents the direction of gene coding). **(F)**: Gene structure map of *Bch10G006400* (*BhHLS1*) (the thick blue rectangle represents the exon region, and the thin rectangle represents the intron region). **(G)**: Comparison of cDNA and protein sequences of *Bch10G006400* (*BhHLS1*) between GX-71 and MY-1 (the red base is the mutation site; *: translation terminated).

Based on the high-density genetic map (total length of 1357.145 cM and an average distance between adjacent markers of 0.002 cM) and phenotypic values in 2021 and 2022, QTL mapping of seed size-related traits was performed using the CIM method. Finally, a total of seven QTLs were identified on 12 chromosomes, including two and five QTLs for SL and SW, respectively (**Figures 3A–D**; **Table 1**). Among them, two QTLs (*qSL10 and qSW10*) were considered major stable QTL as they were detected across both years with phenotypic variation rates (PVE) >10%. *qSL10* and *qSW10* were located on chromosome 10 at 52.23–52.23 and 52.23–52.75 cM (0.52 cM), with maximum limit of detection (LOD) values of 41.57/47.74 and 33.63/34.6, respectively. Notably, *qSL10* and *qSW10* had PVE of 75.3% and 66.6% in 2021 and 80.8% and 73.8% in 2022, respectively (**Table 1**). In addition, their additive effects were 1.4955 and 0.7887 in 2021 and 1.4621 and 0.8403 in 2022, respectively, both of which were positive (**Table 1**). Therefore, it could be concluded that their synergistic genes originated from MY-1 (large seed). Notably, the *qSL10* interval was within the *qSW10* interval (**Figures 3E**), and the PVEs of both were greater than 60%. Hence, we tentatively concluded that the main potentiating genes located on LG10 that regulate SL and SW might be the same gene, and the strong correlation between the two traits (0.94***) supports the same mechanism regulating seed growth and development (**Supplementary Figure 2**).

**Table 1:** Quantitative trait loci (QTLs) for traits related to seed size.

Trait	Year	QTL	Chr	Position interval (cM)	Max LOD	ADD	PVE (%)	Physical interval
SL	2021	*qSL8*	Chr08	91.02-98.83	5.09	-0.3188	3.20%	44446074–48883849 (4.438 Mb)
	2021/2022	*qSL10*	Chr10	52.23–52.23	41.57/47.74	1.4955/1.4621	75.3%/80.8%	15864354–15907809 (0.043 Mb)
SW	2021	*qSW2*	Chr02	69.67-72.79	4.16	-0.198	3.40%	49910840-53964918 (4.054 Mb)
	2022	*qSW8.1*	Chr08	90.42–94.69	3.43	-0.1613	3.00%	44446074–46895204 (2.449 Mb)
	2021	*qSW8.2*	Chr08	90.42-95.21	3.8	-0.1641	3.20%	44446074-48092489 (3.646 Mb)
	2021/2022	*qSW10*	Chr10	52.23–52.75	33.63/34.6	0.7887/0.8403	66.6%/73.8%	15864354–16201714 (0.337 Mb)
	2022	*qSW11*	Chr11	129.68–133.26	3.51	0.176	2.80%	71829002–73319298 (1.490 Mb)

Max LOD, maximum LOD value; ADD, additive effect; PVE, contribution rate.

### 3.4 Identification of candidate genes

QTL mapping analysis revealed that *qSL10* was located within the *qSW10* interval. Based on the unpublished reference genome (GX-19) of the wax gourd and the parental resequencing data, sequence alignment and functional annotation analyses were performed for all candidate genes within the *qSL10* and *qSW10* intervals of the wax gourd. The results showed 12 candidate genes in the *qSW10* region (including three candidate genes in the *qSL10* region: *Bch10G006380*, *Bch10G006390*, and *Bch10G006400*) (**Figure 3E**). Among these, 11 were annotated in the database, whereas the *Bch10G006390* gene had no functional annotation (**Table 2**). From these 12 genes, only CDS of *Bch10G006400*, containing three exons and two introns, had two non-synonymous mutations (**Figures 3F, G**). To further validate the *Bch10G006400* gene from both parents, MY-1 and GX-71, as well as four other wax gourd varieties, namely YSB-1 (large seed), BD (large seed), GM (small seed), and GK (small seed), were used for cloning and sequencing. Sanger sequencing results were consistent with those of resequencing, and the sequences of MY-1, YSB-1, and BD were identical to those of GX-71, GM, and GK. A nonsense mutation and a non-synonymous mutation at the 400 and 808 bp positions of the CDSs, respectively, were observed in GX-71, GM, and GK. Specifically, the guanine (G)-to-uracil (T) mutation at 400 bp was identified to convert the 134^th^ amino acid from glutamic acid to a stop codon, and the guanine (G)-to-adenine (A) mutation at 800 bp was identified to convert the 270^th^ amino acid from alanine to threonine (**Figures 3G**; **Supplementary Figure 3A**; **Supplementary Table S5**). *Bch10G006400* encodes a probable N-acetyltransferase HLS1-like protein (**Table 2**). Analysis using the Batch CD-search tool in NCBI and SMART (https://SMART.Embl-heide-lberg.de/) showed that the protein structure included an Acetyltransf_1 domain (amino acids 57–144), and the nonsense mutation resulted in the deletion of this domain (**Supplementary Figure 3B**). Further functional annotation and sequence alignment analyses showed that *Bch10G006400* (named *BhHLS1*) might be a key gene regulating the seed size in wax gourds.

**Table 2:** MGene annotations of quantitative trait locus (QTL) intervals.

Gene ID	*Nonsynonymous Mutations in Coding Sequences	Physical Location	Gene Annotation
*Bch10G006380*	No	Chr10:15867749–15872889 (-)	BAG family molecular chaperone regulator 4
*Bch10G006390*	No	Chr10:15873955–15874104 (-)	–
*Bch10G006400*	Yes	Chr10:15904540–15906484 (+)	probable N-acetyltransferase HLS1-like
*Bch10G006410*	No	Chr10:15946485–15948259 (+)	serine/threonine-protein phosphatase 7 long form-like protein
*Bch10G006420*	No	Chr10:15983856–15987828 (+)	pentatricopeptide repeat-containing protein At2g30100, chloroplastic
*Bch10G006430*	No	Chr10:15990592–15994011(-)	Inactive receptor-like serine/threonine-protein kinase At2g40270
*Bch10G006440*	No	Chr10:16020178–16021976 (-)	uncharacterized protein At5g39865
*Bch10G006450*	No	Chr10:16061994–16066348 (+)	LRR repeats and ubiquitin-like domain-containing protein At2g30105
*Bch10G006460*	No	Chr10:16067699–16077606 (-)	ubiquitin-activating enzyme E1
*Bch10G006470*	No	Chr10:16102276–16105918 (-)	probable LIM domain-containing serine/threonine-protein kinase DDB_G0287001
*Bch10G006480*	No	Chr10:16142173–16145076 (-)	UDP-glycosyltransferase 87A1-like
*Bch10G006490*	No	Chr10:16173183–16174030 (-)	LOB domain-containing protein 12-like

*The presence or absence of non-synonymous mutations in gene coding sequences. (+) and (-), The coding direction of genes; –, No functional annotation.

### 3.5 qRT-PCR analysis of candidate genes

**Figure 4:**
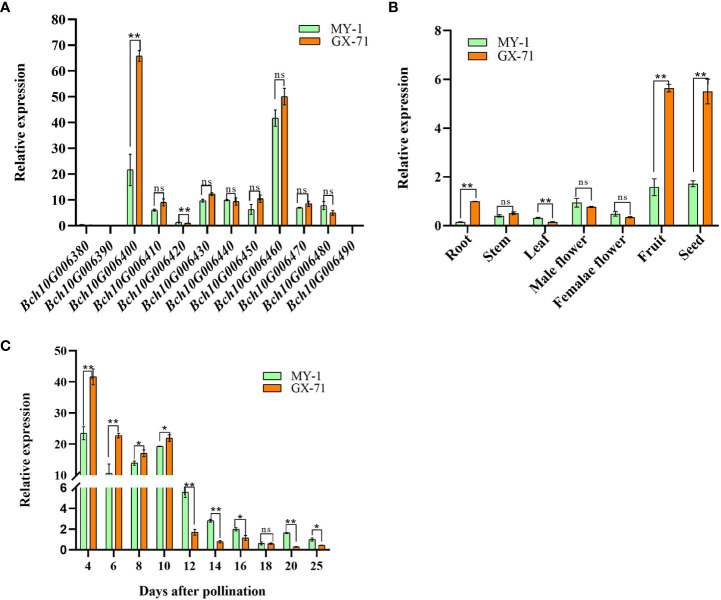
Expression analysis of candidate genes. **(A)**: Expression analysis of 12 genes in seeds between parents 6 days after pollination (DAP) through quantitative real-time PCR. **(B)**: Expression analysis of *BhHLS1* in different tissues of GX-71 and MY-1 based on quantitative real-time PCR. **(C)**: Expression analysis of *BhHLS1* in seeds of GX-71 and MY-1 through quantitative real-time PCR (*P < 0.05, **P < 0.01, ns: no significant difference; each experiment is based on three replicates, and values are presented as the mean ± SE).

To further identify candidate genes, qRT-PCR was used to analyze the spatiotemporal expression of such genes in the parents. Differential expression analysis of all candidate genes within the QTL interval during the rapid-expansion stage (6 DAP) in the parental seeds showed that *Bch10G006400* (*BhHLS1*) and *Bch10G006460* were expressed at high levels. However, *Bch10G006400* (*BhHLS1*) showed significant differences in expression levels between parents, whereas no significant differences were observed for *Bch10G006460*. The expression levels of other genes were relatively low; among these, *Bch10G006380*, *Bch10G006390*, and *Bch10G006490* were basically not expressed in the parent 6 DAP seeds (**Figure 4A**). Therefore, *BhHLS1* may be considered a candidate gene that regulates wax gourd seed size. The differential expression of *BhHLS1* in seeds at different developmental stages (4, 6, 8, 10, 12, 14, 16, 18, 20, and 25 DAP) and among different tissues (root, stem, leaf, female flower, male flower, pulp, and seed) suggested that the expression of *BhHLS1* is specific; the expression was high in the pulp and seeds, and the same expression trend was observed between parents. The expression level was low in other tissues, but there was a significant difference in the roots and leaves between the parents (**Figure 4B**). At different stages of seed development, *BhHLS1* showed similar expression trends between MY-1 and GX-71. Moreover, the expression of *BhHLS1* in the early stages of seed development (4–10 DAP) was significantly higher than that in the middle and later stages of seed development (12–25 DAP) (**Figure 4C**), indicating that *BhHLS1* might play an important role in the regulation of seed size in the early stages of seed development. This expression trend also has a certain correlation with the growth law of wax gourd seeds.

### 3.6 Phylogenetic analysis

**Figure 5:**
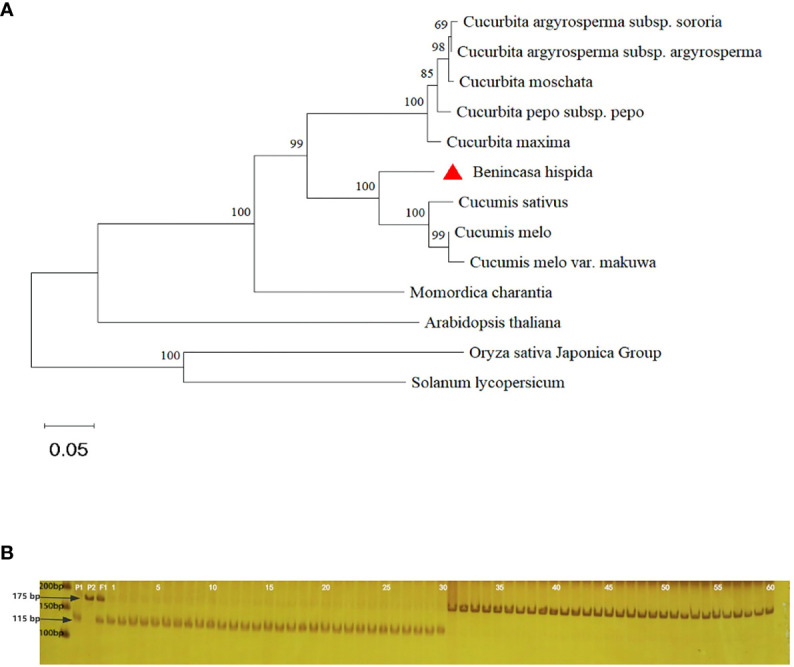
Phylogenetic tree analysis and MAS (molecular marker-assisted selection) test of *BhHLS1*. **(A)**: Phylogenetic tree of BhHLS1 and its homologous proteins. The phylogenetic tree was constructed using MEGA-11 with 1000 bootstrap replications. The numbers at the forks of the tree represent the bootstrap values. **(B)**: The CAPS marker of the first mutation site of *BhHLS1* was used for genotyping wax gourd germplasm resources. P1 and P2 represent MY-1 and GX-7, respectively; 1–28 refer to wax gourds with large seeds, and 29–60 refer to wax gourds with small seeds.

To analyze the relationship among BhHLS1 and other homologous proteins, phylogenetic trees were constructed using the sequences of wax gourd and other Cucurbitaceae plants, as well as *Arabidopsis*, rice, and tomato. The phylogenetic tree showed a close relationship between BhHLS1 and the N-acetyltransferase HLS1-like protein of Cucurbitaceae, which belonged to the same branch as that in melon and cucumber and was relatively distant from that in bitter gourd (**Figure 5A**).

### 3.7 Development of CAPS markers for seed size identification

To further identify candidate genes and apply them to the auxiliary screening of wax gourd seed size in the future, CAPS markers were developed using dCAPS Finder2.0, an online website (http://helix.wustl.edu/dcaps/dcaps.html), based on the single nucleotide polymorphism (SNP) mutation sites found in the coding region of *BhHLS1* (**Supplementary Table S1**). Following PCR amplification, the amplification product of MY-1 could be digested into fragments of 115 and 60 bp with TspR1, but the amplification product of GX-71 could not be digested with this enzyme. The genotypic and phenotypic consistencies among 60 homozygous wax gourd samples with different genetic backgrounds and extreme characteristics, their parents, and F_1_ plants were tested. Among them, bands of 28 large-seed plants (1–28) were consistent with those of MY-1. Meanwhile, of the 32 small-seed plants (29–60), band numbers 29 and 30 were consistent with those of MY-1, whereas those of 31–60 were consistent with the corresponding GX-71 bands; F_1_ had both parental bands, and the coincidence rate of genotype and phenotype was 96.7% (**Figure 5B**).

## 4 Discussion

### 4.1 Mechanism of seed size development in wax gourd

Based on the analysis of the growth curves and SL and SW correlations between MY-1 and GX-71, the SL and SW growth and development curves were single S-shaped, and their changing trends were the same. The fastest growth period was approximately 6–8 DAP, and the SL and SW of GX-71 tended to stabilize earlier than did those of MY-1. The results showed that the initial stage of seed development is the rapid growth period for the seed coat, and is the key period that determines the seed size and morphology. This is consistent with the seed development process and seed size formation stage in watermelons reported by (Guo et al., 2020a). Some studies have shown that functional genes participate in the development of embryos, endosperms, and integuments and control the size of seeds by regulating cell proliferation and elongation. The *ARF2* gene in *A. thaliana* controls seed size by limiting cell proliferation within the capsule through maternal effects (Okushima et al., 2005; Schruff et al., 2006). Moreover, as a suppressor of seed size, *DA1* regulates this process by limiting the cell proliferation period, whereas the *SOD7* gene regulates seed size by inhibiting the integument and cell proliferation (Xia et al., 2013). Additionally, some genes involved in phytohormone regulation control seed size by altering the cell cycle. Brassinolide regulates seed size by altering the embryo and endosperm and regulating the expression of other seed size-related genes (Eun-Ji and Eugenia, 2020). Further, gibberellin, auxin (IAA), and cytokinin directly control seed size by enhancing cell proliferation and affecting embryonic and endosperm development (Du et al., 2017; Hu et al., 2018; Li et al., 2019b; Teshale et al., 2019; Guo et al., 2020b). In this study, histological observations of transverse and longitudinal sections of the mature seeds of the parents revealed differences in the structures of seed coat cells between MY-1 and GX-71. The sclerenchyma of GX-71 was thicker than that of MY-1, and the lignification was severe (**Figures 3A, B**); moreover, there was a significant difference in the numbers and sizes of parenchyma cells (**Figures 3C, D**). Genes that control wax gourd seed size could therefore be involved in regulating cell proliferation, expansion, differentiation, and other cell cycle activities, thus affecting seed size. However, this should be further investigated.

### 4.2 Molecular mechanism through which *BhHLS1* regulates seed size in wax gourd

*BhHLS1* encodes a probable N-acetyltransferase HLS1-like protein belonging to the acetyltransferase (GNAT) protein family (**Table 2**). The acetyltransferase-like protein HOOKLESS1 (HLS1) originates from embryophytes and is a protein similar to the GCN5 acetyltransferase, and HLS1 orthologs are divergent in flowering plants (Wang et al., 2023). By comparing the protein sequences and structural domains of BhHLS1 between the parents, the results showed that the 134th amino acid in GX-71 (small-seed) mutated to a stop codon, which led to the absence of 274 amino acids (**Figures 3G**; **Supplementary Figure 3A**). This mutation further resulted in the deletion of the Acetyltransf_1 domain (**Supplementary Figure 3B**), which is essential for the function of the N-acetyltransferase HLS1-like protein in GX-71. GNAT is a plant histone acetyltransferase, and the GNAT superfamily contains a conserved region that recognizes and binds acetyl-CoA. This structure can transfer the acetyl group of acetyl-CoA to the substrate, thereby catalyzing acetylation (Huang et al., 2014). Recent studies have shown that GNAT proteins are involved in plant seed development. Moreover, Song et al. (2015) identified grain weight trait loci in rice, where *OsglHAT1*, encoding the GNAT protein, was associated with the expansion of the spikelet shell through an increase in cell numbers and acceleration of seed filling, thereby increasing the overall acetylation level of histone H4 and thus improving the grain weight and yield. Further, Dimitra et al. (2010) showed that three genes of the GNAT/MYST family are differentially expressed at different stages during barley seed development, as well as among varieties exhibiting different seed sizes and weights, and that they participate in the development of barley seeds. There is also increasing evidence that hormone signaling is involved in controlling seed size (Garcia et al., 2003; Ishimaru et al., 2013; Li et al., 2019b). However, as a scaffold protein, HLS1 is associated with various transcriptional regulatory factors and is considered a molecular hub for integrating exogenous and endogenous signals (Wang et al., 2023). *HLS1* is an ethylene-responsive gene necessary for apical hook development and auxin asymmetrical distribution (Anne et al., 1996; Wang et al., 2023). Based on *HLS1* mutants, these genes were found to be involved in regulating the ABA-response pathway by regulating ABI5 expression (Liao et al., 2016). Moreover, *HLS1* could be involved in the negative feedback regulation of IAA homeostasis by controlling *GH3* genes (Ohto et al., 2006). In addition, *HLS1* interacts with jasmonic acid and gibberellin (An et al., 2012; Song et al., 2014; Zhang et al., 2014). In summary, *HLS1* plays a key role in plant growth and development.

In a spatiotemporal expression analysis of *BhHLS1* (**Figure 4C**), the expression level of *BhHLS1* was significantly different between parents. It is further explained that *BhHLS1* is a key gene in regulating seed growth. In addition, the expression of *BhHLS1* in GX-71 was significantly higher at 4–10 DAP than that in MY-1, but this was reversed at 12–25 DAP (**Figure 4C**). It is noteworthy that the seed size was basically unchanged in the late stages of seed development (**Figures 1F, G**), meanwhile, the expression of *BhHLS1* in parents was significantly lower than that in the early stages of seed development (**Figure 4C**). This was in line with the growth pattern of wax gourd seeds. The differential expression of *BhHLS1* in different stages of parent seeds may be related to the complex molecular regulatory mechanism of *BhHLS1.* This is a question worth exploring, and we will further study the function of *BhHLS1*.

Based on the results of this study, it is speculated that the structural deletion of BhHLS1 protein changes the protein function, and *BhHLS1* might affect the seed coat cell number and cell size during the development of wax gourd seeds through complex molecular regulatory mechanisms, ultimately contributing to the changes in seed size.

### 4.3 Molecular marker-assisted breeding for seed size in wax gourd

Progress has been made in developing and applying molecular markers of fruit-related traits in wax gourd (Cheng et al., 2021; Ma et al., 2021; Huang et al., 2022; Wu et al., 2022b). In this study, a CAPS marker based on an exon G/T mutation in *BhHLS1* was developed for the testing of genotypic and phenotypic concordance using 60 extreme materials with an accuracy of 96.7% (**Figure 5B**) and can thus be used for future marker-assisted breeding. Some studies have shown that QTLs regulating seed size are generally pleiotropic. For example, the *EVM0009818* controls SL and SW in melon (Zhang et al., 2022), and *qSS14* simultaneously regulates four yield-related traits (100-SW, SL, SW, and ST) in soybean (Yuan et al., 2023). In our study, the pleiotropic gene *BhHLS1* simultaneously regulated two seed size-related traits (SL and SW), which was also confirmed by the high correlation between SL and SW (0.94***) (**Supplementary Figure 2**). However, owing to the immature genetic transformation system of wax gourds, there are some obstacles in exploring the molecular mechanism through which *BhHLS1* regulates seed size. Our findings provide a theoretical basis for further research on this mechanism, in addition to providing a theoretical reference for seed size-regulating genes in other species of Cucurbitaceae that are closely related to *BhHLS1*. In future studies, combined with optimized genetic transformation methods, the key role of *BhHLS1* in regulating wax gourd seed size will be further validated using gene editing and overexpression techniques. Nevertheless, the results of this study are significant for wax gourd agriculture in terms of the genetic improvement in seed size, verification of gene functions, and cultivation of specific germplasm resources.

## Data availability statement

The datasets presented in this study can be found in online repositories. The names of the repository/repositories and accession number(s) can be found below: CNSA accession number: CNP0004715, https://db.cngb.org/search/?q=CNP0004715.

## Author contributions

WY: Data curation, Validation, Writing – original draft, Formal Analysis. PW: Methodology, Writing – review & editing. TL: Writing – review & editing, Data curation, Investigation. LN: Data curation, Investigation, Writing – review & editing. ZKC: Investigation, Writing – review & editing, Software. LS: Investigation, Writing – review & editing. WB: Investigation, Writing – review & editing. YD: Investigation, Writing – review & editing. ZHC: Investigation, Writing – review & editing. ZL: Writing – review & editing, Methodology, Conceptualization.
