# Supplementary material for: Fine mapping of the major gene BhHLS1 controlling seed size in wax gourd (Benincasa hispida)
Source: Front Plant Sci. 2023 Sep 29;14:1266796. doi: 10.3389/fpls.2023.1266796 (PMC10570438; doi:10.3389/fpls.2023.1266796)
Supplement: Supplementary file 1 [file DataSheet_1.docx]

**Supplementary Material**

**Supplementary Table S1**. Primers used in this study.

| **Primer ID** | **Forward sequence (5′****—3′)** | **Reverse sequence (5′—3′)** | | **Application** |
| --- | --- | --- | --- | --- |
| KL-40 | ATGGGGTTTGTTATTCGATGCT | TTATACTTCTCTTGGGTCTACAAAGAGAG | CDS of *Bch10G006400* | |
| *CAC* | AAGTTTGTGGTTGAGGCTGTTG | GAGCGAACTCCTTCTTGAGTGA | qRT-PCR for *CAC* | |
| qPCR-38 | ATGGCTCTTACCGTCATCAAATC | TATGCAACCACTCGTCGTTCTC | qRT‐PCR for *Bch10G006380* | |
| qPCR-39 | CAATGGCCCAATTAAAGAAATTG | TGTTTGTGATAAAAAAAGAGGACGA | qRT‐PCR for *Bch10G006390* | |
| qPCR-40 | GTGGTTGGTGTTATTCAAGGCTC | AATCTTCCAAACGACGGACG | qRT‐PCR for *Bch10G006400* | |
| qPCR-41 | CGCACATCTGGATTTTATGGG | CAGGTAACCCCAACAACACTTCT | qRT‐PCR for *Bch10G006410* | |
| qPCR-42 | TTCTCTCAAGAAGGGAGAGATTCAT | CCATTTCCCAATACAAGGAGATAAC | qRT‐PCR for *Bch10G006420* | |
| qPCR-43 | CGAGACTCTGTTTTTCTCTGAACAA | GTTCAGTATCACGACTTTCCCATC | qRT‐PCR for *Bch10G006430* | |
| qPCR-44 | CGATGGTCCAGATTCCTGATAAG | GACCATGCTCTAAGCAAGGAATT | qRT‐PCR for *Bch10G006440* | |
| qPCR-45 | AACTTCAGCGAGAGTGCTTGATT | CAGACTCCCAAGTGAAGAAGGC | qRT‐PCR for *Bch10G006450* | |
| qPCR-46 | TGAAGAAGGTGTGGTTGTAGAAGG | ACAGCAGCAACGTTACTGTTAGAAT | qRT‐PCR for *Bch10G006460* | |
| qPCR-47 | GTGTTGATGAGGACCCAGGATAG | TTCTTAGACCCCTCAGTTCTCGTAG | qRT‐PCR for *Bch10G006470* | |
| qPCR-48 | CTCAAAAATCCCAACATTCTCATC | AGAGTCTCAACGGGAGCTTCC | qRT‐PCR for *Bch10G006480* | |
| qPCR-49 | ATAAGATCTTCGGTGCTAGCAATG | TTCGTTTTGTAGAAACGAGATTGTC | qRT‐PCR for *Bch10G006490* | |
| CAPS-40 | TCGTCCGTCGTTTGGAAGAT | TGATGAAGAGATTAAGAGAGGCATG | MAS test | |

**Supplementary Table S2**. Accession numbers of amino acid sequences downloaded from NCBI.

| **Species** | **Accession** |
| --- | --- |
| *Benincasa hispida* | XP_038902314.1 |
| *Cucumis melo* | XP_008465276.1 |
| *Cucumis sativus* | XP_011658419.2 |
| *Cucurbita argyrosperma subsp. sororia* | KAG6605401.1 |
| *Cucurbita maxima* | XP_023007288.1 |
| *Cucumis melo var. makuwa* | KAA0067323.1 |
| *Cucurbita argyrosperma subsp. argyrosperma* | KAG7035352.1 |
| *Cucurbita pepo subsp. pepo* | XP_023532249.1 |
| *Cucurbita moschata* | XP_022947633.1 |
| *Momordica charantia* | XP_022148434.1 |
| *Arabidopsis thaliana* | NP_180570.1 |
| *Oryza sativa Japonica Group* | XP_015628406.1 |
| *Solanum lycopersicum* | XP_004233456.1 |

**Supplementary Table S3**. MAS testing in 60 *Benincasa hispida* inbred lines and parents lines and F_1_ using CAPS-40.

| **Number** | **Inbred line** | **Genotype *** | **Phenotype** | **Number** | **Inbred line** | **Genotype *** | **Phenotype** |
| --- | --- | --- | --- | --- | --- | --- | --- |
| P1 | MY-1 | A | Large seed | 30 | GK-2 | A | Small seed |
| P2 | GX-71 | B | Small seed | 31 | 7-3 | B | Small seed |
| F_1_ | F_1_ of MY-1×GX-71 | H | The middle type | 32 | TY-2 | B | Small seed |
| 1 | BD | A | Large seed | 33 | 7-2 | B | Small seed |
| 2 | YSB-1 | A | Large seed | 34 | LQ | B | Small seed |
| 3 | DF-1 | A | Large seed | 35 | B-1 | B | Small seed |
| 4 | LZZ01 | A | Large seed | 36 | 2-1 | B | Small seed |
| 5 | F-3 | A | Large seed | 37 | 2-2 | B | Small seed |
| 6 | TK-1 | A | Large seed | 38 | LP | B | Small seed |
| 7 | HF-7 | A | Large seed | 39 | KF-4 | B | Small seed |
| 8 | M-1 | A | Large seed | 40 | HJJG | B | Small seed |
| 9 | GD-2 | A | Large seed | 41 | TL-1 | B | Small seed |
| 10 | TK-2 | A | Large seed | 42 | HCJG | B | Small seed |
| 11 | KM-1 | A | Large seed | 43 | 2-3 | B | Small seed |
| 12 | HXK-1 | A | Large seed | 44 | TY-5 | B | Small seed |
| 13 | HXK-4 | A | Large seed | 45 | GH-1 | B | Small seed |
| 14 | F-5 | A | Large seed | 46 | LZ | B | Small seed |
| 15 | F-2 | A | Large seed | 47 | 7-4 | B | Small seed |
| 16 | F-4 | A | Large seed | 48 | GF | B | Small seed |
| 17 | HF-2 | A | Large seed | 49 | KX-2 | B | Small seed |
| 18 | F-1 | A | Large seed | 50 | GK-1-3 | B | Small seed |
| 19 | F-7 | A | Large seed | 51 | TM-1 | B | Small seed |
| 20 | F-6 | A | Large seed | 52 | 7-1 | B | Small seed |
| 21 | GD-2 | A | Large seed | 53 | TM-2 | B | Small seed |
| 22 | YO-6 | A | Large seed | 54 | 7-6 | B | Small seed |
| 23 | HXK-3 | A | Large seed | 55 | 7-5 | B | Small seed |
| 24 | HT-7 | A | Large seed | 56 | KX-2 | B | Small seed |
| 25 | RSDG | A | Large seed | 57 | LV-6 | B | Small seed |
| 26 | TK-4 | A | Large seed | 58 | LV-2 | B | Small seed |
| 27 | HXK-2 | A | Large seed | 59 | YF-4 | B | Small seed |
| 28 | KM-3 | A | Large seed | 60 | KF-2 | B | Small seed |
| 29 | TY-3 | A | Small seed |  |  |  |  |

* A representatives genotype of male parent (Large seed), B representatives genotype of female parent (Small seed), H representatives Heterozygote.

**Supplementary Table S4.** Descriptive statistics of seed size in recombinant inbred line (n = 105) population developed from cross MY-1 x GX-71.

| **Trait#** | **Year** | **Parents** | |  | **RIL** | | | | |  |
| --- | --- | --- | --- | --- | --- | --- | --- | --- | --- | --- |
|  |  | **MY-1(Mean)** | **GX-71(Mean)** |  | **Minimum** | **Maximum** | **Range** | **Mean** | **Standard deviation** | |
| **SL (mm)** | 2021 | 10.022 | 7.128 |  | 6.229 | 11.455 | 5.227 | 9.033 | 0.156 | |
|  | 2022 | 9.983 | 6.983 |  | 6.636 | 11.595 | 4.959 | 9.199 | 0.153 | |
| **SW (mm)** | 2021 | 6.523 | 4.254 |  | 3.611 | 6.859 | 3.248 | 5.150 | 0.087 | |
|  | 2022 | 6.204 | 4.027 |  | 3.580 | 7.027 | 3.447 | 5.265 | 0.088 | |

# SL-Seed length; SW-Seed width; RIL: recombinant inbred line

**Supplementary Table S5**. Sequence alignment of MY-1 and GX-71.

| **Type** | **Sequence** |
| --- | --- |
| CDS Sequence of MY-1 | ATGGGGTTTGTTATTCGATGCTACGAAGAAAGTCAATTATCAGATAAAGCTCAAGTTATAGACCTTGAACGAAGATGTCAAATTGGCCAATCAAAACGTGTCTTTCTCTTCACTGACAATTTGGGTGACCCCATTTGTAGAATACGTAATAGTCCCATGTATAAAATGCTGGTTGCTGAGTGGGACAAGGAAGTGGTTGGTGTTATTCAAGGCTCTATAAAAGCGGTTTTTTTGACTGCTCATAAACCGCCACCGCCCGGTTTGGTGGTTAAAGTGGGCTACATTCTTGGCTTGAGAGTGGCGCCACCGTATCGCCGCCGTGGGATTGGCTCCGGCCTCGTCCGTCGTTTGGAAGATTGGTTTGTTTCTAATGATGTTGATTACTGTTGCATGGCCACTGAGAAAGATAATCATGCCTCTCTTAATCTCTTCATCAATAATTTAAGGTACATAAAGTTTAGAACAGGAAGAATCTTAGTAGACCCAGTAAGAAATCGTCCATACAATATCAATTCATCAGAAATCAACATTCAAAAGCTAAAAATAGAAGAAGCAGAAGCAATATACAAAAAACACATGGCCTCAACAGAGTTCTTCCCCAAAGACATAAAAAGCATATTGAAAAACAAGCTGAGCTTAGGGACATGGATGGCAAATTTCAAACAACCGCCATGGTTGTCGCCGTCGACCGCTGTCGGCGGAAACAGGCAGATTACAACGAGCAGCTGGGCCATTGCAAGTCTATGGAATAGTGGGGAAGTTTTCAAGCTAAGGCTAGGAAAAGCACCATTTCCATGGCTTATTTACGCAAAAAGTTTAAAAATTATGGATAAAATTTTGCCTTGCTTTAAGCTTGTTTTGGTGCCTGATTTTTTCAAGCCATTTGGGTTTTATTTTGTTTATGGATTGCACCATGAAGGCCCTTTTTCTGAGAGATTGGTTGGAGCTTTGTGCAAATTTGTGCATAATGTGGCATTGAAGAATAATTCAAGGGATAGTTGTAAAGCTATTGTTACAGAGATTGGTGGTGATGAAGATGATGAGCTGAAAATGGAGATTCCTCATTGGAAATTGCTATCATGTTATGAAGATTTTTGGTGCATAAAGTCCTTGAGAAATAATAATATTAGTAATGATAATGATCATGATCATGATCATCATATATTGGAATGGACAAATGCCCCACCTAATAGAACTCTCTTTGTAGACCCAAGAGAAGTATAA |
| CDS Sequence of GX-71 | ATGGGGTTTGTTATTCGATGCTACGAAGAAAGTCAATTATCAGATAAAGCTCAAGTTATAGACCTTGAACGAAGATGTCAAATTGGCCAATCAAAACGTGTCTTTCTCTTCACTGACAATTTGGGTGACCCCATTTGTAGAATACGTAATAGTCCCATGTATAAAATGCTGGTTGCTGAGTGGGACAAGGAAGTGGTTGGTGTTATTCAAGGCTCTATAAAAGCGGTTTTTTTGACTGCTCATAAACCGCCACCGCCCGGTTTGGTGGTTAAAGTGGGCTACATTCTTGGCTTGAGAGTGGCGCCACCGTATCGCCGCCGTGGGATTGGCTCCGGCCTCGTCCGTCGTTTGGAAGATTGGTTTGTTTCTAATGATGTTGATTACTGTTGCATGGCCACTTAGAAAGATAATCATGCCTCTCTTAATCTCTTCATCAATAATTTAAGGTACATAAAGTTTAGAACAGGAAGAATCTTAGTAGACCCAGTAAGAAATCGTCCATACAATATCAATTCATCAGAAATCAACATTCAAAAGCTAAAAATAGAAGAAGCAGAAGCAATATACAAAAAACACATGGCCTCAACAGAGTTCTTCCCCAAAGACATAAAAAGCATATTGAAAAACAAGCTGAGCTTAGGGACATGGATGGCAAATTTCAAACAACCGCCATGGTTGTCGCCGTCGACCGCTGTCGGCGGAAACAGGCAGATTACAACGAGCAGCTGGGCCATTGCAAGTCTATGGAATAGTGGGGAAGTTTTCAAGCTAAGGCTAGGAAAAGCACCATTTCCATGGCTTATTTACACAAAAAGTTTAAAAATTATGGATAAAATTTTGCCTTGCTTTAAGCTTGTTTTGGTGCCTGATTTTTTCAAGCCATTTGGGTTTTATTTTGTTTATGGATTGCACCATGAAGGCCCTTTTTCTGAGAGATTGGTTGGAGCTTTGTGCAAATTTGTGCATAATGTGGCATTGAAGAATAATTCAAGGGATAGTTGTAAAGCTATTGTTACAGAGATTGGTGGTGATGAAGATGATGAGCTGAAAATGGAGATTCCTCATTGGAAATTGCTATCATGTTATGAAGATTTTTGGTGCATAAAGTCCTTGAGAAATAATAATATTAGTAATGATAATGATCATGATCATGATCATCATATATTGGAATGGACAAATGCCCCACCTAATAGAACTCTCTTTGTAGACCCAAGAGAAGTATAA |
| Protein sequence of MY-1 | MGFVIRCYEESQLSDKAQVIDLERRCQIGQSKRVFLFTDNLGDPICRIRNSPMYKMLVAEWDKEVVGVIQGSIKAVFLTAHKPPPPGLVVKVGYILGLRVAPPYRRRGIGSGLVRRLEDWFVSNDVDYCCMATEKDNHASLNLFINNLRYIKFRTGRILVDPVRNRPYNINSSEINIQKLKIEEAEAIYKKHMASTEFFPKDIKSILKNKLSLGTWMANFKQPPWLSPSTAVGGNRQITTSSWAIASLWNSGEVFKLRLGKAPFPWLIYAKSLKIMDKILPCFKLVLVPDFFKPFGFYFVYGLHHEGPFSERLVGALCKFVHNVALKNNSRDSCKAIVTEIGGDEDDELKMEIPHWKLLSCYEDFWCIKSLRNNNISNDNDHDHDHHILEWTNAPPNRTLFVDPREV* |
| Protein sequence of GX-71 | MGFVIRCYEESQLSDKAQVIDLERRCQIGQSKRVFLFTDNLGDPICRIRNSPMYKMLVAEWDKEVVGVIQGSIKAVFLTAHKPPPPGLVVKVGYILGLRVAPPYRRRGIGSGLVRRLEDWFVSNDVDYCCMAT* |

**Note:** In Table S5, the highlighted font indicates the variation of the CDS sequence and amino acid sequence alignment of the two parents.

**
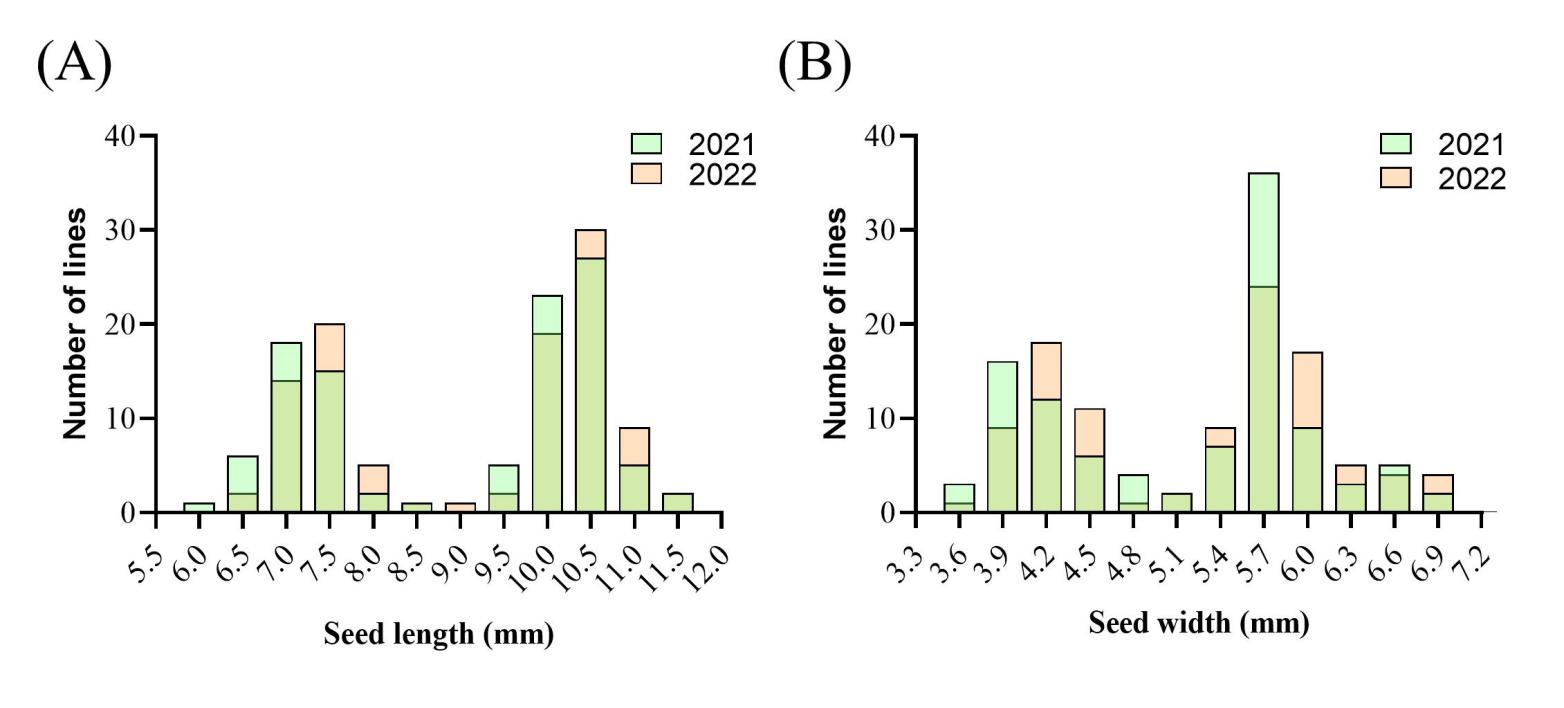
**

**Supplementary Figure 1.** Frequency distribution of seed length (A) and seed width (B) in the RIL population derived from a cross between ‘MY-1’ and ‘GX-71’ across 2 years (2021 and 2022).

**
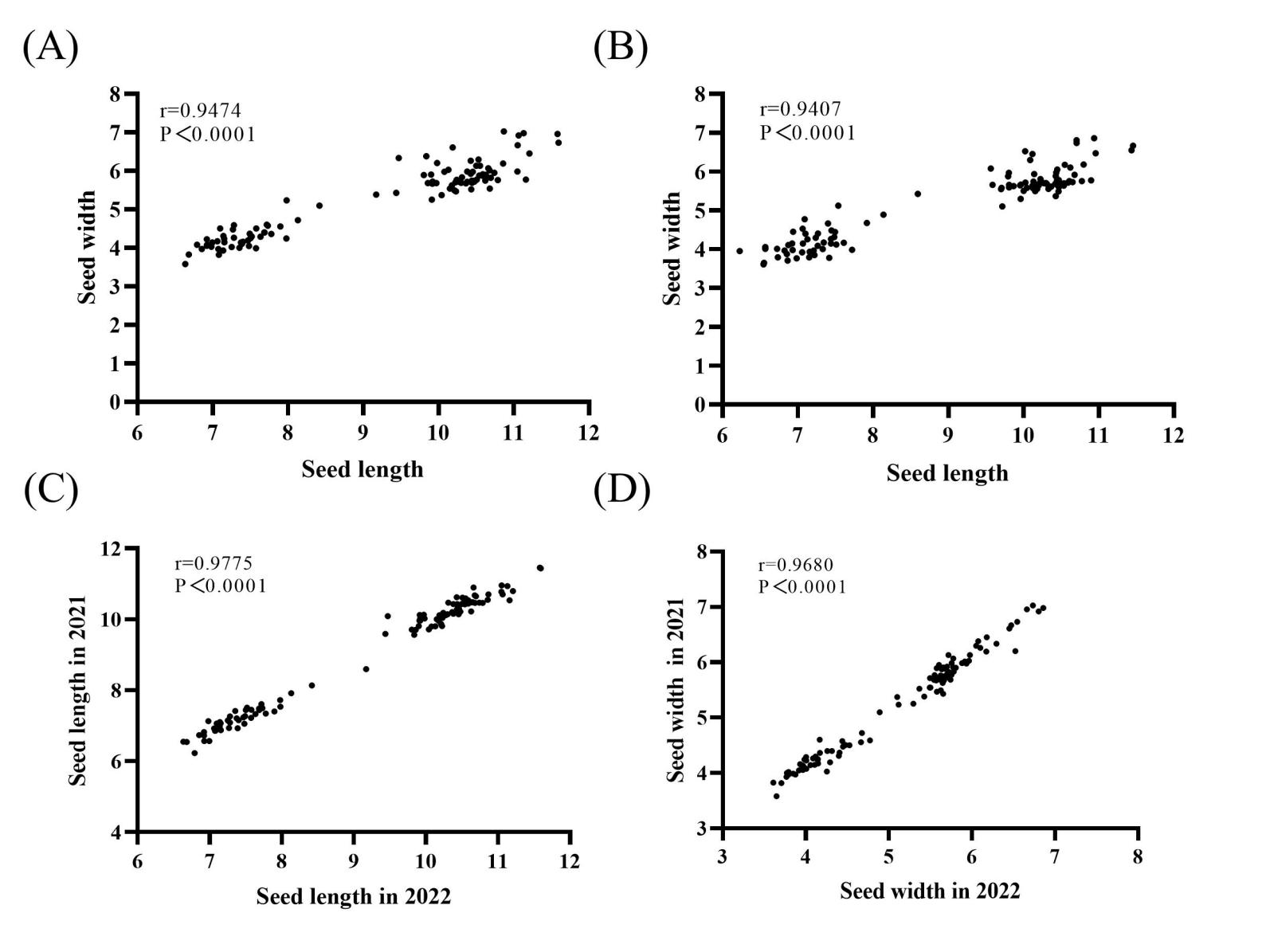
**

**Supplementary Figure 2.** Correlation analysis of seed length and seed width in 2021 (A) and 2022 (B), correlation analysis of seed length in 2021 and 2022 (C) and seed width in 2021 and 2022 (D).

**
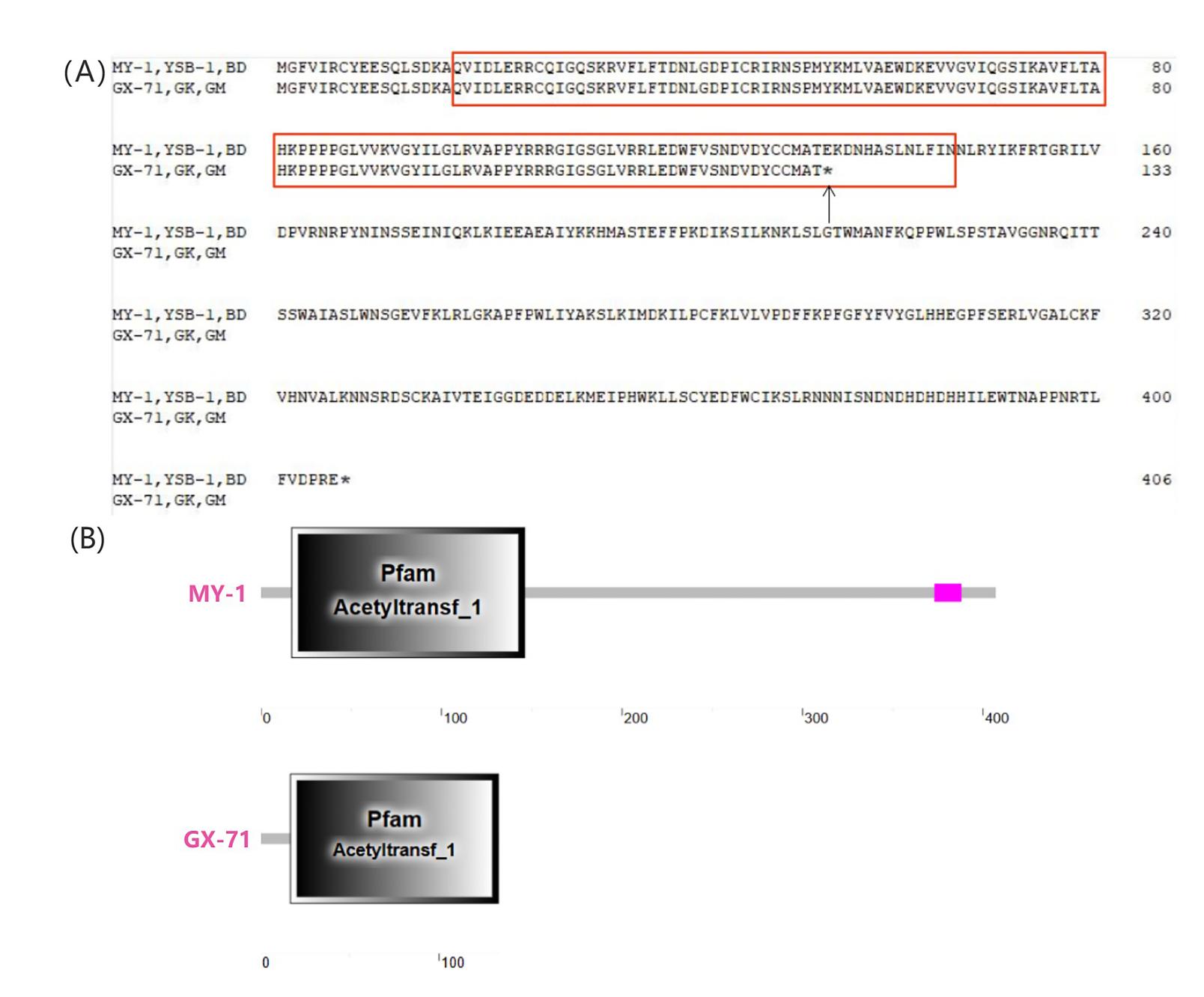
**

**Supplementary Figure 3.** Comparison of BhHLS1 amino acid sequences among six wax gourd materials, and protein structure analysis between MY-1 and GX-71. **(A)**: Comparison of amino acid sequences encoded by the *BhHLS1* gene among six materials (MY-1, YSB-1, and BD are varieties with large seeds; GX-71, GK, and GM are varieties with small seeds; the red framed region is the Acetyltransf_1 protein domain, the site indicated by the arrow is the SNP mutation site, and * indicates the termination of translation). **(B)**: Changes in the protein domains of BhHLS1 between MY-1 and GX-71.
